# FREE TESTOSTERONE, SEX HORMONE BINDING GLOBULIN, AND INCIDENT FRAILTY IN MEN WITH HIV

**DOI:** 10.1093/geroni/igae098.2782

**Published:** 2024-12-31

**Authors:** Jenny Pena Dias, Kathryn C Fitzgerald, Michelle Shardell, Steven Wolinsky, Jordan Lake, Susan L Koletar, Shezhad Basaria, Todd Brown

**Affiliations:** Johns Hopkins University, Baltimore, Maryland, United States; Johns Hopkins University, Baltimore, Maryland, United States; University of Maryland School of Medicine, Baltimore, Maryland, United States; Northwestern Medicine, Chicago, Illinois, United States; McGovern Medical School, Houston, Texas, United States; The Ohio State University, Columbus, Ohio, United States; Brigham and Women’s Hospital, Boston, Massachusetts, United States; Johns Hopkins, Baltimore, Maryland, United States

## Abstract

**Background:**

Older men with HIV (MWH) have a higher prevalence of frailty than matched men without HIV, but the underlying mechanisms are unclear. Testosterone (T) contributes to higher muscle (mass, strength), and it binds to sex hormone-binding globulin (SHBG), which regulates testosterone bioavailability to the tissues. Aging MWH experience increase in circulating SHBG and decline in circulating free T concentrations. We hypothesized that these hormonal changes may contribute to the development of frailty in MWH.

**Methods:**

Free T and SHBG were measured in 306 non-frail MWH (129 robust and 177 prefrail) who had at least one frailty assessment at a semi-annual follow-up visit in the Multicenter AIDS Cohort Study. Marginal structural models were used and adjusted for covariates: age, race, education, comorbidity burden, BMI, smoking status, alcohol use, HIV parameters and loss to follow-up.

**Results:**

Mean age at baseline was 55±5 years, (median 58 years, Q1=55, Q3=63); follow up frailty assessments visits was 8.5(SD:2.5) years. MWH with low free T (< 50 pg/ml) were more likely to become frail compared to those with free T ≥50 pg/ml (HR,1.58 (95%CI:1.12, 2.24); p-value=0.03). Low free T was also significantly associated with transition from prefrailty to frailty, [< 50 pg/ml: 47%; ≥50 pg/ml:34%], a difference of 12.4% (95% CI:2.9,24.3%). In contrast, SHBG concentrations were not associated incident frailty or transition to frailty.

**Conclusion:**

Low free T, but not SHBG, predicted incident frailty in men with HIV at high risk of developing frailty, in particular those in the prefrail state.

